# Supplementary material for: University students’ health-related quality of life and its determinants. Results from a cross-sectional survey during the COVID-19 pandemic
Source: PLoS One. 2025 Jan 6;20(1):e0310378. doi: 10.1371/journal.pone.0310378 (PMC11703069; doi:10.1371/journal.pone.0310378)
Supplement: Attachment — Submitted filename: Comment to Author - PLOS ONE.pdf [file pone.0310378.s002.pdf]

Comment

Healthy minds in healthy bodies? University students' health-related quality of life and its determinants. Results from a cross-sectional survey during the COVID-19 pandemic.

Abstract: To enhance the abstract, a brief background is added in the first paragraph, setting the context for the investigation. The conclusion is recommended to be refined based on specific results, as the current conclusion appears too general. Furthermore, it is suggested to include implications of the research in the last paragraph to provide readers with a clearer understanding of the broader significance of the study.

Introduction: The overall structure of the introduction is well-organized, but it is advised to include a definition of the quality of life (QoL) of students to provide a better understanding for readers. Additionally, in lines 87–89, the impact of COVID-19 on the QoL of students should be clearly stated, supported by information from previous reports or research. Lines 86–87 could benefit from further clarification on the purpose and benefits of the research to strengthen the research gap. Lastly, the rationale behind focusing on the physical and mental aspects of QoL and the choice of SF-12 as the study instrument should be explained.

Sampling: The sampling methodology should be clearly stated, including whether it involves purposive sampling, snowball sampling, or another method. In lines 105–107, it is suggested to calculate the sample size based on the university population, considering the proportion of students from each subject or degree.

Discussion: The discussion section requires improvement by providing more in-depth insights into the results, relating them to existing theories, and considering the context of the university. Specific suggestions include explaining the gender differences in the MCS (result in line 161 - 162), the reasons behind the QoL differences between students with and without migration backgrounds (result in line 163 - 164), and the factors contributing to variations in PCS and MCS scores among different majors (Line 165 – 167). Additionally, explanations for the impact of the pandemic on student health-related QoL (Line 211 – 213), as well as any difference with previous research, should be addressed.

Furthermore, it is recommended to discuss the implications of the research, particularly in terms of how the findings can be applied to enhance the physical and mental well-being of university students.

Conclusion: The conclusion's highlight is deemed too general, and it is suggested to refine it based on specific results, the research context, and the implications of the study. This will provide a stronger and more insightful conclusion that resonates with the study's objectives and contributions.
