# Supplementary material for: University students’ health-related quality of life and its determinants. Results from a cross-sectional survey during the COVID-19 pandemic
Source: PLoS One. 2025 Jan 6;20(1):e0310378. doi: 10.1371/journal.pone.0310378 (PMC11703069; doi:10.1371/journal.pone.0310378)
Supplement: Attachment — Submitted filename: Comments to Authors.pdf [file pone.0310378.s004.pdf]

Comments

University students’ health-related quality of life and its determinants. Results from a cross-sectional survey during the COVID-19 pandemic.

Abstract

- Please use consistent decimal formatting throughout the manuscript. For instance, on line 38, change '63% were female' to '63.0% were female,' because most of the decimal formatting in your manuscript follows the pattern of having one decimal point.

Discussion

- Line 257: I recommend changing the topic to something specific and related to your discussion, such as sociodemographic characteristics and university students’ quality of life, because your current topic might not be clear to readers.

Conclusion

- Lines 342 – 354: I recommend linking the implications of the study in the discussion part within the related topic rather than the conclusion part. After that, summarize the implications in the conclusion part without citing them in the text. For example, in Line 342 - 346, Universities could use these findings to improve their students’ well-being in different ways: First, our findings could inform measures that take the unequal distribution of quality of life into account and specifically address particularly burdened groups of students. This could entail the establishment or increase of peer support groups, which has previously been shown to be effective for international students in the USA [53] and for Black women studying at predominantly white institutions [54]. This aspect, which is highlighted in the discussion regarding the relevance of migration background for mental health, should be addressed in the final or a related paragraph of the discussion. Afterward, you can summarize these aspects in the conclusion. For example, in the conclusion, the research highlights the importance of recognizing differences in students' quality of life and the need for customized support to help marginalized groups. One effective solution is strengthening peer support systems, which have proven helpful in various situations. These results underscore the significance of addressing issues such as migration background when discussing mental health among college students.
