# Supplementary material for: University students’ health-related quality of life and its determinants. Results from a cross-sectional survey during the COVID-19 pandemic
Source: PLoS One. 2025 Jan 6;20(1):e0310378. doi: 10.1371/journal.pone.0310378 (PMC11703069; doi:10.1371/journal.pone.0310378)
Supplement: Attachment — Submitted filename: Feb 2024.docx [file pone.0310378.s001.docx]

**Comments**

**Title**

**You need to revise the title**

**Abstract**

**Please describe little bit about sociodemographic**

**Introduction**

**From the beginning of paragraph, you should focus on mental issues in Covid 19 pandemic. The explanation about the background of the study looked biased and too wide.**

**Method**

**Sampling**

**You should explain the sampling method because I can’t see it in the manuscript.**

**Questionnaire**

**QoL SF 12,..which language does this original questionnaire use? If in English, you should do translation to Germany language then do pilot study again to get the Cronbach alpha.**

**Results**

**Why did you not do analysis bivariate between sociodemographic and the QoL Score?**

**Because I do not see any meaning here, you just presented the scoring result and we do not know the correlation with the sociodemographic because you should know to have mental problem, a lot of contributing factors for that.**
