# Supplementary material for: University students’ health-related quality of life and its determinants. Results from a cross-sectional survey during the COVID-19 pandemic
Source: PLoS One. 2025 Jan 6;20(1):e0310378. doi: 10.1371/journal.pone.0310378 (PMC11703069; doi:10.1371/journal.pone.0310378)
Supplement: Attachment — Submitted filename: Response to reviewers_final.docx [file pone.0310378.s003.docx]

Dear Sir or Madam,

We thank the editor for the opportunity to revise our article and we thank the reviewers for their valuable comments and suggestions. Overall, we feel that the revision further improved the article and we hope that our revisions meet the reviewers’ concerns.

Below, we comment in detail how we implemented each suggestion:

**Reviewer 1:**

1. Title: “You need to revise the title”

We removed the first part of the title to make it shorter and more concise.

2. Abstract: “Please describe little bit about sociodemographic”

Thank you for this suggestion, which greatly improves the abstract! We added the most important sociodemographic information to the abstract (as much as we could, respecting the word limit of 300 words).

3. Introduction: “From the beginning of paragraph, you should focus on mental issues in Covid 19 pandemic. The explanation about the background of the study looked biased and too wide.”

Thank you for this feedback. We added more information on the relevance of the pandemic and its effects on university students, and hope that this clarifies the background of our research question.

4. Method: “Sampling. You should explain the sampling method because I can’t see it in the manuscript.”

The explanations concerning our sampling procedure are outlined on lines 117 to 124. Initially, we aimed to construct a sampling frame from the university’s list of students’ email addresses and employ an address-based proportionate sampling taking the respondents’ subject into account.

Since the university denied us access to this list, we had to employ the strategy described in the manuscript, where we tried to invite as many students as possible using mailing lists and student organizations to distribute invitations. The resulting recruitment strategy is probably best described as “convenience sampling”. We added this information to the manuscript.

5. Method: “Questionnaire. QoL SF 12, which language does this original questionnaire use? If in English, you should do translation to Germany language then do pilot study again to get the Cronbach alpha.”

We used the German version of the SF-12, which has been validated already in 1998 (Gandek et al. 1998) and has been extensively used since then (see also Morfeld et al. 2011). Therefore, a separate validation study was not needed. We added this information to the manuscript (lines 128-131).

6. Results: “Why did you not do analysis bivariate between sociodemographic and the QoL Score? Because I do not see any meaning here, you just presented the scoring result and we do not know the correlation with the sociodemographic because you should know to have mental problem, a lot of contributing factors for that.”

We agree that it is interesting to see how QoL is associated with particular sociodemographic characteristics and that bivariate analyses would have been one way to look at this question.

Meanwhile, the stratified analyses that we conducted and described in lines 181 to 194 are another way of answering this question (and in some ways more suitable than bivariate regression analyses, because the presentation of mean values for different sociodemographic subgroups allows for better comparison with the literature, where the reporting of mean values for the SF-12 dimensions is very common).

Hereby, we find that women, non-Germans, migrants and law students have lower QoL than their respective comparison group and the sample’s average. In addition, we present the stratified analyses in Table 3.

We therefore believe that the manuscript and Table 3 already answer the question how sociodemographics and QoL are associated. Nevertheless, if we misunderstood this comment and it aims at something else, we are happy to revisit this question again.

**Reviewer 2:**

1. Abstract: “To enhance the abstract, a brief background is added in the first paragraph, setting the context for the investigation. The conclusion is recommended to be refined based on specific results, as the current conclusion appears too general. Furthermore, it is suggested to include implications of the research in the last paragraph to provide readers with a clearer understanding of the broader significance of the study.”

Thank you for these suggestions to improve the abstract. We added an introduction at the beginning of the abstract and revised the conclusion so that it is more closely connected with our findings. Together with the added information on the demographics of the sample (in reaction to reviewer 1), we now utilize the word limit of 300 words and therefore cannot elaborate more on the study’s implications, even though we agree that this would have been beneficial.

2. Introduction: “The overall structure of the introduction is well-organized, but it is advised to include a definition of the quality of life (QoL) of students to provide a better understanding for readers. Additionally, in lines 87–89, the impact of COVID-19 on the QoL of students should be clearly stated, supported by information from previous reports or research. Lines 86–87 could benefit from further clarification on the purpose and benefits of the research to strengthen the research gap. Lastly, the rationale behind focusing on the physical and mental aspects of QoL and the choice of SF-12 as the study instrument should be explained.”

Thank you for this positive feedback! We are happy to further improve the introduction and included your suggestions at two different places:

First, we added an explanation that defines QoL and explains why this is an important proxy for both overall well-being but also predictive for a vast array of medical outcomes.

Second, we elaborated a bit more on the impact of COVID on the situation of students and briefly draft previous studies. Hereby the research gap and the rational for our study become clearer now.

Lastly, we revised the methods section and included an explanation on the choice of the SF12 and explain that we chose this instrument primarily because it is since many years established in quality of life research and therefore offers the option for comparison with other target groups but also with studies from before the pandemic.

3. Sampling: “The sampling methodology should be clearly stated, including whether it involves purposive sampling, snowball sampling, or another method. In lines 105–107, it is suggested to calculate the sample size based on the university population, considering the proportion of students from each subject or degree.”

The sampling strategy is – as discussed in the manuscript – one of the study’s limitations: Initially, we aimed to construct a sampling frame from the university’s list of students’ email addresses and employ an address-based proportionate sampling taking the respondents’ subject into account.

Unfortunately, university denied us access to this list. Therefore, we had to employ the strategy described in the manuscript, where we tried to invite as many students as possible using mailing lists and student organizations to distribute invitations. The resulting recruitment strategy is probably best described as “convenience sampling”. We added this information to the manuscript.

With respect to the second part of the reviewer’s comment, we are not sure if we understand it correctly. Our approach is exploratory in nature (as opposed to confirmatory), therefore we chose not to perform any sample size calculations beforehand. In our view, this is in line with current guidelines and established practice (e.g. Wasserstein and Lazar 2016 or Greenland et al. 2016). In case we misunderstood the reviewer’s suggestion, we are happy to comply with it after further explanation.

4. Discussion: “The discussion section requires improvement by providing more in-depth insights into the results, relating them to existing theories, and considering the context of the university. Specific suggestions include explaining the gender differences in the MCS (result in line 161 - 162), the reasons behind the QoL differences between students with and without migration backgrounds (result in line 163 - 164), and the factors contributing to variations in PCS and MCS scores among different majors (Line 165 – 167). Additionally, explanations for the impact of the pandemic on student health-related QoL (Line 211 – 213), as well as any difference with previous research, should be addressed.”

Thank you for these detailed suggestions for improvements of the discussion section. We are happy to take this opportunity to increase the clarity and rigor of the discussion. To that end, we now structure the discussion by using subheadings and elaborate on each of the topics in a separate paragraph. In addition, we added more references to previous studies and give more space to theoretical explanations of our findings, especially with respect to the importance of migration background as a predictor of quality of life.

5. “Furthermore, it is recommended to discuss the implications of the research, particularly in terms of how the findings can be applied to enhance the physical and mental well-being of university students.”

AND

“The conclusion's highlight is deemed too general, and it is suggested to refine it based on specific results, the research context, and the implications of the study. This will provide a stronger and more insightful conclusion that resonates with the study's objectives and contributions.”

Thank you for this suggestion. We agree that the conclusion was too general. We revised it and hereby tried to connect it more closely with our findings and also be more specific in terms of practical implications.
